# Mechanistic Study of Common Non-Nucleoside Reverse Transcriptase Inhibitor-Resistant Mutations with K103N and Y181C Substitutions

**DOI:** 10.3390/v8100263

**Published:** 2016-09-23

**Authors:** Ming-Tain Lai, Vandna Munshi, Meiqing Lu, MeiZhen Feng, Renee Hrin-Solt, Philip M. McKenna, Daria J. Hazuda, Michael D. Miller

**Affiliations:** Department of Antiviral Research, Merck Research Laboratories, West Point, PA 19486, USA; vandna_a_munshi@merck.com (V.M.); meiqing_lu@merck.com (M.L.); meizhen_feng@merck.com (M.F.); my3jacks@comcast.net (R.H.-S.); philip_mckenna@merck.com (P.M.M.); daria_hazuda@merck.com (D.J.H.); michael_miller1@merck.com (M.D.M.)

**Keywords:** resistance mechanism, K103N, Y181C, non-nucleoside reverse transcriptase inhibitor, NNRTI-associated mutations

## Abstract

Non-nucleoside reverse transcriptase inhibitors (NNRTIs) are a mainstay of therapy for human immunodeficiency type 1 virus (HIV-1) infections. However, their effectiveness can be hampered by the emergence of resistant mutations. To aid in designing effective NNRTIs against the resistant mutants, it is important to understand the resistance mechanism of the mutations. Here, we investigate the mechanism of the two most prevalent NNRTI-associated mutations with K103N or Y181C substitution. Virus and reverse transcriptase (RT) with K103N/Y188F, K103A, or K103E substitutions and with Y181F, Y188F, or Y181F/Y188F substitutions were employed to study the resistance mechanism of the K103N and Y181C mutants, respectively. Results showed that the virus and RT with K103N/Y188F substitutions displayed similar resistance levels to the virus and RT with K103N substitution versus NNRTIs. Virus and RT containing Y181F, Y188F, or Y181F/Y188F substitution exhibited either enhanced or similar susceptibility to NNRTIs compared with the wild type (WT) virus. These results suggest that the hydrogen bond between N103 and Y188 may not play an important role in the resistance of the K103N variant to NNRTIs. Furthermore, the results from the studies with the Y181 or Y188 variant provide the direct evidence that aromatic π–π stacking plays a crucial role in the binding of NNRTIs to RT.

## 1. Introduction

The current treatment for human immunodeficiency type 1 virus (HIV-1) infected patients, known as highly active anti-retroviral therapy (HAART), typically consists of three or more drugs with complementary mechanisms of action [1]. Non-nucleoside reverse transcriptase (RT) inhibitors (NNRTIs) constitute a cornerstone in this therapy and are commonly used in combination with inhibitors that suppress HIV-1 replication via different mechanisms. However, during the course of HIV-1 treatment with an NNRTI-containing regimen, the coupling of the error-prone HIV-1 RT enzyme—with the absence of an exonuclease proofreading activity—results in emerging NNRTI-associated resistant viruses. Moreover, a single mutation often causes cross-resistance to drugs in the same class. Therefore, a new generation of NNRTIs is needed with more resilience against current prevalent mutations. To facilitate the development of better NNRTIs, it is important to understand the resistance mechanisms of the prevalent mutations; especially those arising from the K103N and Y181C substitutions in RT.

The K103N mutant is the most common mutation associated with NNRTIs, which is estimated to be present in 40%–60% of NNRTI-resistant viruses [2,3,4]. The mutant displays significant resistance to the first generation NNRTIs; such as efavirenz (EFV), nevirapine (NVP), and delavirdine (DLV). Amino acid residue K103 is located near a putative entrance to the NNRTI binding pocket (NNRTIBP). Examination of the structures of the K103N RT/NNRTI complexes shows that the K103N substitution induces only minor positional adjustments of the NNRTIs and residues in the binding pocket—when compared with structures of wild type (WT) RT/NNRTI complexes. Therefore, based on the X-ray structures, NNRTIs bind to the RT mutant in a conservative mode rather than through major rearrangements [5,6].

A hypothesis has been proposed to elucidate the resistance mechanism from the K103N substitution [7]. Comparison of the structures between the unliganded WT and K103N RT reveal a network of hydrogen bonds in the K103N mutant that is not present in the WT enzyme. These hydrogen bond networks contribute to the stabilization of the closed-pocket form of the enzyme, which could interfere with inhibitor accessibility to the NNRTIBP. In particular, a hydrogen bond in the unliganded K103N RT (but not in WT RT) was observed between N103 and Y188. Since the Y188 and N103 are located at the wing1 and wing2 positions of the binding pocket, respectively, a hydrogen bond between them would pose a hurdle for inhibitors to access the binding pocket, giving rise to resistance to many NNRTIs [7].

Alternatively, it is hypothesized that resistance is attributed to changes in hydrophobic and electrostatic interactions, which are induced by the replacement of lysine (K) with asparagine (N). The long aliphatic side chain of K103 (four carbons) may engage in close hydrophobic contact with wing2, based on the butterfly-like conformation of NNRTIs [8]. In contrast, asparagine has a much shorter aliphatic side chain in comparison to lysine (1 versus 4). Therefore, the K103N substitution will reduce the affinity to NNRTIBP, if NNRTIs rely on the hydrophobic interactions for their potencies. Furthermore, since the amide group of asparagine is at close proximity with wing2 of NNRTIs, the oxygen atom in the amide group may cause electrostatic repulsion with a highly electronegative group in NNRTIs; such as the trifluoromethyl group in EFV and the nitrogen in the pyridine ring of NVP. Therefore, as proposed by Lindberg, et al., hydrophobic and electrostatic interactions may play a major role in conferring resistance to NNRTIs [9,10].

Another prevalent NNRTI-resistant mutation is Y181C substitution, which is present in 15%–25% of NNRTI-resistant viruses [2,3,4]. This mutation confers a high degree of resistance to both NVP and DLV and a moderate resistance to rilpivirine RPV [11]. A relatively rare mutation of Y188L was also identified in viruses from patients who have failed with NNRTI-containing regimens. Y188L was shown to be highly resistant to first-generation NNRTIs such as EFV and NVP. Crystal structures of WT RT—with the first generation compounds—show extensive stacking interactions of Y181 and Y188 with the aromatic rings of the inhibitors [12,13,14,15,16,17,18]. As a result, it is hypothesized that the resistance of Y181C and Y188L RT mutations is attributed to the loss of π–π stacking of aromatic rings between the residues and NNRTIs. Such interactions are significant contributors to the binding energy of first-generation NNRTIs [19,20]. However, up to date, no direct evidence has been provided to prove the importance of aromatic π–π stacking. 

The purpose of this study was to investigate the mechanism of resistance conferred by the two most prevalent NNRTI mutations with K103N and Y181C substitutions. To determine if the hydrogen bond between N103 and Y188 contributes to the resistance of K103N substitution, the tyrosine (Y) at 188 was replaced with phenylalanine (P). No hydrogen bond between F188 and N103 can be formed with the Y188F/K103N substitutions, as there is no hydroxyl group in phenylalanine. In addition, substitution of K103 with alanine (A) or glutamic acid (E) was designed to evaluate the impacts of the length of aliphatic side chain on the binding of NNRTIs to the NNRTIBP. Moreover, Y181F, Y188F, and Y181F/Y188F mutant RTs were generated to directly test the significance of π–π stacking on the NNRTIs’ affinity to the binding pocket. The experiments were conducted with purified recombinant RTs, as well as mutant viruses containing the mutations of interest. The results suggested that the hydrogen bonding between N103 and Y188 and the hydrophobic interactions may not play a major role in blocking NNRTIs binding to the pocket. Instead, electrostatic interaction may play a more important role in conferring the resistance of the K103N mutant/RT to the first-generation NNRTIs. In addition, this study also provides the direct evidence of the critical role of aromatic π–π stacking between Y181 (or Y188) and NNRTIs in the binding of NNRTIs to NNRTIBP. 

## 2. Materials and Methods 

### 2.1. Sources of Materials

Full-length WT and two mutant RT proteins (K103N and Y181C) were expressed in *Escherichia coli* BL21 (DE3) cells and purified as described previously [21]. The t500 RNA template was made by IBA BioTAGnology (Göttingen, Germany). The biotinylated DNA primer was made by Integrated DNA Technology (IDT, Coralville, IA, USA). DNase I and isopropyl-β-d-thiogalactopryranoside (IPTG) were purchased from Invitrogen (Carlsbad, CA, USA). The R8 virus was a kind gift from Christopher Aiken (Vanderbilt University, Nashville, TN, USA). The Ruthenylated dUTP (Ru-dUTP) was custom-made by Midland Certified Reagents Company (Midland, TX, USA). 3-[(3-cholamidopropyl)dimethylammonio]-1-propanesulfonate hydrate (CHAPS) and1,4-dithiothreitol (DTT) were bought from Sigma-Aldrich (St. Louise, MD, USA), The rotor JA-12 was from Beckman Coulter (Indianapolis, IN, USA). Amicon Ultra membranes were obtained from Millipore (Billerica, MA, USA). The electrochemiluminescence (ECL) detector M-384 and the streptavidin-coated magnetic beads were purchased from BioVeris (Gaithersburg, MD, USA). The culture medium (RPMI-1640) and Dulbecco’s modified Eagle medium (DMEM)/10% fetal bovine serum (FBS) were bought from Gibco (Carlsbad, CA, USA). The Versene/ethylenediaminetetraacetic acid (EDTA) was ordered from BioWhitaker (Walkersville, MD, USA). BL21 CodonPlus (DE3)-RIL competent cells and The QuikChange SDM kit was ordered from were acquired from Agilent Technologies (Santa Clara, CA, USA). The 384-well microplate was purchased from Falcon (Franklin Lake, NJ, USA). The pET-Duet competent cells were obtained from Novagen (Madison, WI, USA). SoftLink avidin resin was purchased from Promega (Madison, WI, USA). β-galactosidase was from New England Biolabs (Ipswich, MA, USA). The VICTOR luminometer was bought from PerkinElmer (Waltham, MA, USA).

### 2.2. Cloning, Expression, and Purification of Biotinylated RT Mutants 

Biotinylated RT (bio-RT) was generated as described previously [22]. Briefly, plasmid pTD101—which was derived from pET-Duet-1 and contained HIV-1 protease cDNA—was inserted into the first multiple cloning site (MCS) without tags. HIV-1 RT cDNA was inserted into the second MCS with AviTag cDNAs (amino acid sequence of GLNDIFEAQKIEWHE at the 3′ end) and was transformed into BL21 CodonPlus (DE3)-RIL competent cells. These were grown on lysogeny broth (LB) agar plates supplemented with 100 mg/L ampicillin at 37 °C overnight. A streak of cells was used to inoculate 100 mL of LB medium, containing 100 mg/L ampicillin, and the culture was grown overnight in a shaker at 37 °C and 225 rpm overnight. The next morning, cells were harvested at 10,000 rpm (Beckman Coulter JA-12 rotor) for 5 min and resuspended in 100 mL fresh media with 100 mg/L ampicillin; 10 mL of which was inoculated into 1 L of LB medium with 100 mg ampicillin. The resulting culture was grown in a shaker at 25 °C and 225 rpm until the optical density at a wavelength of 600 nm (OD_600_) reached 0.6–0.7. Induction of protein expression was initiated by addition of 1 mM IPTG and incubation was continued at 25 °C and 225 rpm for 2 h. Cells were harvested by centrifugation at 20,000 rpm (Beckman Coulter JA-12 rotor) for 10 min. Cell pellets were resuspended (with stirring for 30 min) in 100 mL of cold buffer (buffer A: 50 mM Tris pH 7.8; 60 mM KCl; 6 mM MgCl_2_; 0.025% CHAPS; 5% glycerol; 1 mM DTT,specified in 2.1; DNase I (200 units); and one tablet complete EDTA-free protease inhibitor cocktail), and sonicated using a half inch flat tip at 30% power in 10-s bursts/rests, for a total processing time of 5 min. The lysates were centrifuged twice in a Beckman Coulter JA-12 rotor at 12,000 rpm at 4 °C for 15 min. The resultant supernatant was applied (according to manufacturer’s instructions) to a 10 mL pre-equilibrated/blocked SoftLink avidin column at a gravity flowrate. After completing column loading, the columns were washed with 15 column volumes (CV) of buffer A at a gravity flowrate, and then step eluted using equilibration buffer plus 5 mM biotin in two CV fractions. The eluted fractions were analyzed by sodium dodecyl sulfate polyacrylamide gel electrophoresis (SDS-PAGE) and western blot. The bio-RT was normally eluted from the column with 5 to 6 mL of 5 mM biotin in buffer A. RT fractions were pooled and dialyzed twice against 5 L of buffer A (without protease inhibitors) to remove excess biotin. The dialyzed pool was then concentrated using 10 kDa Amicon Ultra membranes. The protein concentration was determined by amino acid analysis.

### 2.3. HIV-1 Reverse Transcriptase Biochemical Assay 

The ECL reverse transcriptase biochemical assay was performed based on the protocol described previously [23]. Briefly, HIV-1 RT enzyme (10 pM) was combined with an inhibitor or dimethyl sulfoxide (DMSO) (10%) in an assay buffer (50 mM Tris-HCl, pH 7.8), followed by incubation at room temperature for 30 min. The addition of a biotinylated template/primer substrate (5 nM) and deoxyribose nucleosides triphosphate (dNTPs) (0.6 μM of dATP, dGTP, and dCTP, and 20 nM Ru-dUTP) initiated the polymerization reaction. The reactions were continued for 90 min at 37 °C, followed with the addition of 10 μL of 1 N NaOH to terminate the reaction. The resulting solution was incubated at room temperature for an additional 30 min, and then neutralized with 10 µL of 1 N HCl. Streptavidin-coated magnetic beads (80 µg/mL) were added to capture the product and the unreacted substrate for detection. Quantification of the product was determined based on the ECL signal, which was measured by ECL instrument M-384.

### 2.4. Generation of Mutant Viruses

Mutant viruses containing the desired mutation(s) were generated via the introduction of the respective mutation(s) into the WT HIV-1 strain HXB2 backbone, using the QuikChange site-directed mutagenesis (SDM) kit (Agilent, Wilmington, DE, USA). The resulting proviral DNA constructs were used to transfect 293T cells. Culture supernatants containing the SDM-derived mutant viruses were collected, concentrated, and titered prior to the infection experiments [24].

### 2.5. HIV-1 Single Cycle Replication Assay

Hela P4/R5 cells containing the β-galactosidase gene—with the long terminal repeat (LTR) as the promoter—were detached from the plate using Versene^TM^/EDTA, and placed in a 384-well plate at a density of 1000 cells per well. Cells were plated in the presence of a 40-µL medium containing DMEM/10% FBS/1% penicillin/streptomycin. After incubation at 37 °C and 5% CO_2_ overnight, the 20-µL supernatant was removed from the cells and was added to a 384-well plate containing the inhibitor, in order to generate a mixture containing a 2× concentration of viruses and inhibitors(the number of viruses added was dependent on the infectivity of the individual virus). The resulting mixture (20 µL) was added back to the cells described above (in a 20 µL medium), resulting in a 1× concentration of both the virus and the inhibitor. The mixture was then incubated for an additional 48 h. Viral replication gave rise to the expression of β-galactosidase in infected cells. RT inhibitor activity was evaluated using 10 serial-drug concentrations. The level of viral replication was assessed by the addition of a β-galactosidase substrate at a 1:50 ratio. After incubation for 60–90 min, the mixtures were read on a VICTOR luminometer.

Susceptible viruses produce low levels of β-galactosidase activity in the presence of antiviral drugs, whereas viruses with reduced susceptibility produce higher levels of β-galactosidase activity. Therefore, drug susceptibility was measured by comparing the amount of β-galactosidase activity produced in the presence of the RT inhibitor to the amount of β-galactosidase activity produced in the absence of the RT inhibitor. 

Data were analyzed by plotting the percent inhibition of β-galactosidase activity versus a log_10_ drug concentration. Inhibition curves were fitted to the data by a four-parameter fitting algorithm, and were used to calculate the concentration of drugs required to inhibit viral replication by 50% (also known as half maximal effective concentration, EC_50_).

## 3. Results

### 3.1. Characterization of Bio-RT Variants

**Figure 1:**
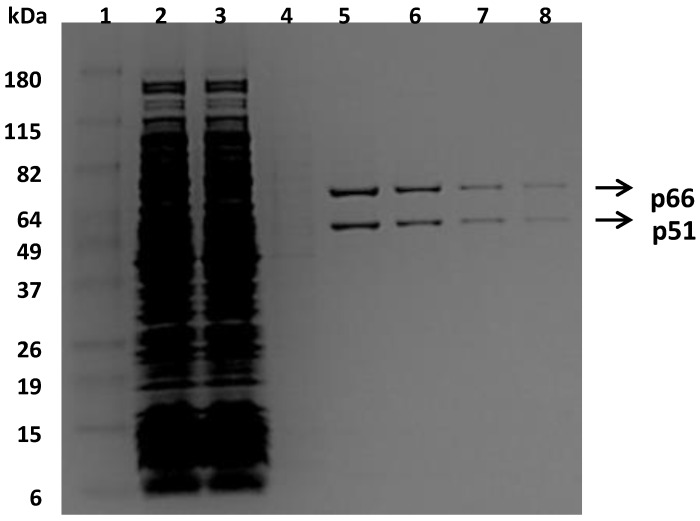
Sodium dodecyl sulfate polyacrylamide gel electrophoresis (SDS-PAGE) analysis of fractions collected from the purification of bio-reverse transcriptase (bio-RT) using the avidin affinity column. Lane 1, molecular weight marker; Lane 2, flow-through fraction; Lanes 3–4, washing fractions; Lanes 5–8, eluted fractions.

To facilitate the purification of recombinant RTs, a small peptide (GLNDIFEAQKIEWHE), which is a substrate of biotin ligase, was added to the C-terminal end of a p66 subunit for the incorporation of the biotin group. This approach enabled the purification of the heterodimer bio-RT into one step by using an avidin affinity column, as shown in Figure 1. The bio-RTs (WT, K103N, or Y181C) were tested for their susceptibility to various NNRTIs and compared with respective non-biotinylated RTs (non-bio-RTs). The results suggested that the potencies with bio-RTs—determined in this study—are similar to the potencies reported previously with non-bio-RTs [25]. For example, the inhibitory potency (IC_50_, which is the inhibitor concentration causing 50% inhibition of the desired activity) of EFV is 0.42 nM and 0.52 nM versus WT RT and corresponding bio-RT, respectively. The IC_50_ of NVP is 137 nM and 140 nM versus WT RT and corresponding bio-RT, respectively. Therefore, incorporation of the biotin and the small peptide on the C-terminal end does not interfere with the function of RT, and the differences in the susceptibility of RT variants and mutant viruses to NNRTIs can be ascribed to the impacts of the specific substitutions in the RTs.

### 3.2. Inhibitory Potency of NNRTIs against Mutant RTs

To study the resistance mechanism of K103N, several different substitutions were generated at the position of 103 in RT. As aforementioned, one of the hypotheses on the resistance mechanism of K103N is that asparagine at 103 can form a hydrogen bond with the hydroxyl group of tyrosine at position 188; thus blocking the entrance of NNRTIBP and interfering with the binding of NNRTIs (such as EFV, NVP, and DLV) to the pocket. To test this hypothesis, RT with double substitutions K103N/Y188F was generated to prevent the formation of a hydrogen bond between the two residues (due to the lack of hydroxyl group in phenylalanine). If the hydrogen bond is responsible for the resistance to NNRTIs, NNRTIs that were impacted by the K103N mutation should regain their potency against the Y188F/K103N RT. In addition, the K103A and K103E RTs were also produced to investigate if hydrophobic or electrostatic interactions between NNRTIs and K103 side chains play an important role in the binding of NNRTIs to the pocket.

The role of aromaticity at positions 181 and 188 of RT—in the binding of RT to NNRTIs—was also investigated. The tyrosine residue at both positions was mutated to phenylalanine, to give Y181F and/or Y188F to directly test the impact of the aromaticity at the positions on the potency of NNRTIs. If the aromaticity of the residues (thus π–π stacking) play a major role in the binding of NNRTIs to the NNRTIBP, it is expected that the Y181F and Y188F RTs should maintain, or even increase, the susceptibility of the RTs to NNRTIs—compared with the WT RT—because the mutation(s) enhance the aromaticity of the residues due to the lack of hydroxyl group.

**Figure 2:**
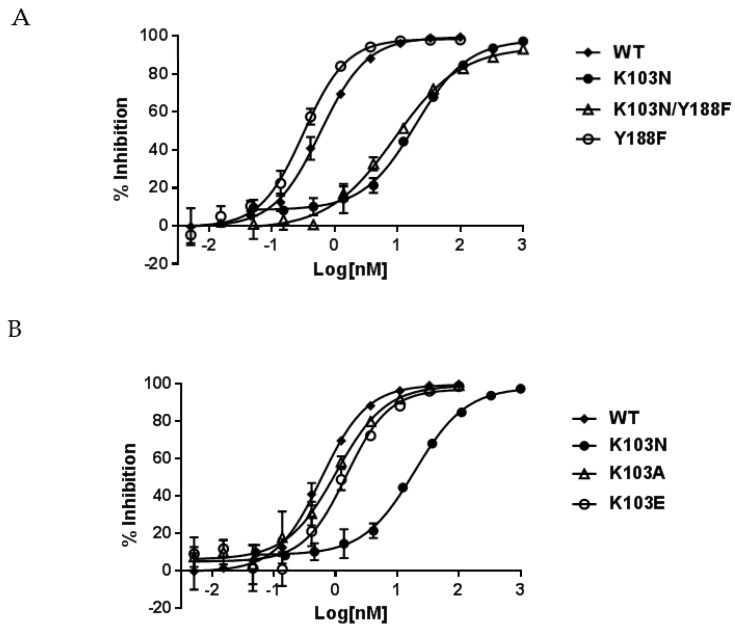
Inhibition curves of efavirenz (EFV) against K103 variants and/or Y188F RTs. (**A**) Wild type (WT) RT, K103N RT, Y188F RT, and K103N/Y188F RT; (**B**) WT RT, K103N RT, K103E RT, and K103A RT. The error bars are in means ± standard deviation with greater than three replicates.

The inhibitory potency of NNRTIs against these mutant bio-RTs is summarized in Table 1. The left panel of Table 1 indicates that both K103N and K103N/Y188F RTs conferred a similar level of resistance to NNRTIs. For example, EFV displayed IC_50_s of 17.0 nM and 10.5 nM against K103N and K103N/Y188F RTs, respectively. When compared to the potency of 0.52 nM against WT RT, these represent 33- and 20-fold of resistance, respectively. In addition, EFV showed an approximately two-fold better potency (IC_50_ of 0.32 nM) in inhibiting Y188F RT (Table 1, right panel) than WT RT. Therefore, this suggests that the resistance conferred by K103N/Y188F RT is ascribed to K103N, not Y188F. The representative inhibition curves of EFV versus the mutant RTs are shown in Figure 2A. K103N is also highly resistant to NVP and DLV, with IC_50_s of 8100 nM and 2600 nM representing 58- and 27-fold of resistance, respectively. Comparable levels of resistance are also conferred by K103N/Y188F RT versus NVP and DLV—with IC_50_ of 10,033 nM and 1290 nM—which are 72- and 13-fold of resistance, respectively. Similarly, the Y188F substitution did not reduce the affinity of NVP and DLV to the mutant RT. This is consistent with the findings from EFV studies. Taken together, these results indicate that the resistance from the K103N substitution may not be attributed to the hydrogen bond between N103 and Y188. When K103A and K103E RTs were evaluated for their susceptibility to the NNRTIs, K103A and K103E RTs only exhibited low levels of resistance to the NNRTIs (<3-fold versus EFV and <5-fold with NVP and DLV) (Table 1, right panel). The representative inhibition curves with EFV versus the mutant RTs are shown in Figure 2B.

**Figure 3:**
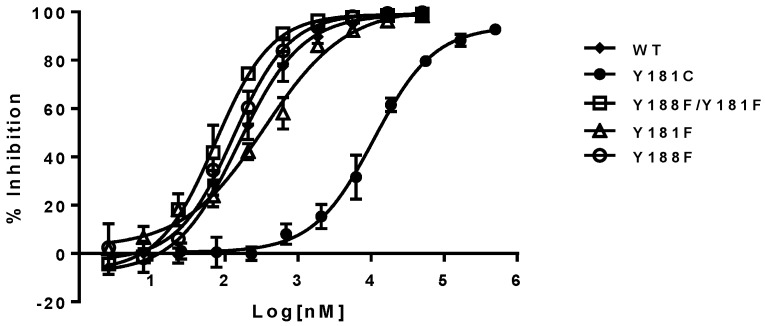
Inhibition curves of nevirapine (NVP) against Y181 and Y188 RT variants. The error bars are in means ± standard deviation with more than three replicates.

The inhibitory potency of NNRTIs versus RTs with phenylalanine at 181, 188, and both 181/188 positions—instead of tyrosine—is summarized in Table 1 (right panel). It appears that both Y181F and Y188F RTs display similar susceptibility to the NNRTIs, with an approximately two-fold higher susceptibility compared to WT RT. In contrast, RT, with the double substitutions of Y181F/Y188F, increased its sensitivity from 3- to 8-fold to all NNRTIs tested in this study. As expected, Y181C RT is highly resistant to NVP and DLV, but not EFV. On the contrary, Y188L RT displayed significant resistance to all of the three NNRTIs, which is consistent with previous reports [25,26,27]. The representative inhibition curves with NVP versus the mutants are shown in Figure 3. 

### 3.3. Antiviral Activity of NNRTIs against Mutant Viruses Containing RT Mutations

**Figure 4:**
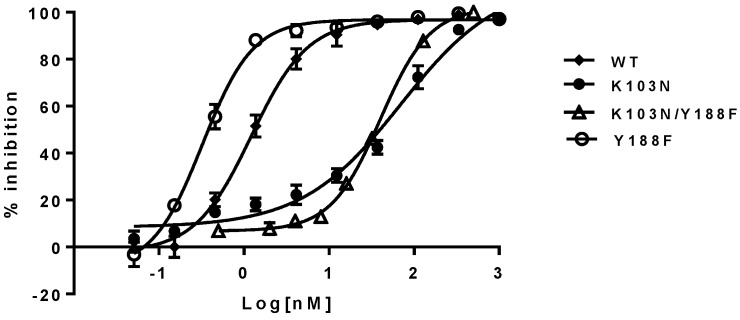
Inhibition curves of EFV against K103 and/or Y188F mutants. The error bars are in means ± standard deviation with more than three replicates.

To investigate if the findings observed in a biochemical assay can be recapitulated in a cell-based assay, the respective mutant viruses were generated by introducing the mutations described above into the RT region of the HIV-1 genome. Their susceptibility to the NNRTIs (EFV, NVP, and DLV) was evaluated with a single-cycle replication assay. The results are summarized in Table 2. As shown in the left panel of Table 2, the EC_50_s of the NNRTIs against the Y188F/K103N and K103N mutant viruses are within a 2- to 3-fold difference. For example, EFV displayed EC_50_ of 69 nM and 40 nM against K103N and K103N/Y188F mutants, respectively. These represent 58- and 33-fold of resistance when compared with 1.2 nM against the WT virus. Yet the Y188F virus appears to be four-fold more susceptible to the NNRTIs than the WT virus (Table 2, right panel). These results are consistent with the trend observed in the biochemical assay, which, again, suggest that the hydrogen bond between N103 and Y188 may not contribute to the resistance of K103N to the NNRTIs. The representative inhibition curves of EFV against the mutants are shown in Figure 4. 

K103A and K103E mutant viruses displayed similar susceptibility to the NNRTIs compared with the WT virus, as opposed to a low level of resistance to the NNRTIs observed in biochemical assay. K103A and K103E substitutions did not confer high levels of resistance to NNRTIs as K103N did in both biochemical and cell-based assays. Therefore, the resistance of K103N may not be due to the lack of hydrophobic interactions between NNRTIs and K103N, owning to shorter aliphatic side chain of asparagine (1 versus 4) because both K103A and K103E possess only one and two carbon aliphatic side chains, respectively.

**Figure 5:**
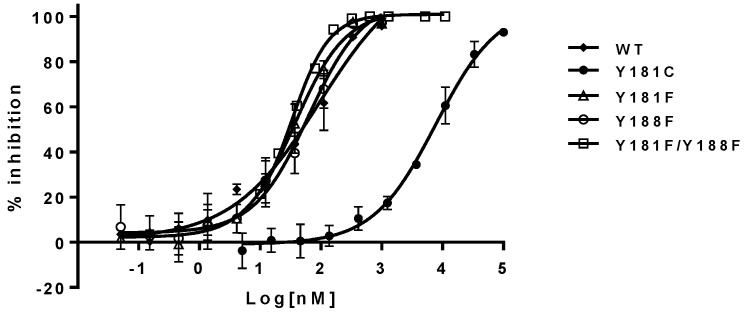
Inhibition curves of NVP against Y181 and Y188 mutants. The error bars are in means ± standard deviation with more than three replicates.

The impacts of aromaticity (at positions 181 and/or 188) on the susceptibility of the mutants to NNRTIs are summarized in the right panel of Table 2. The Y188F/Y181F mutant virus was from approximately 3- to 6-fold more susceptible to all NNRTIs than the WT virus. These observations are also consistent with the biochemical assay results. In addition, the Y181F virus also appears to be three-fold more susceptible to all the NNRTIs compared with the WT virus. The Y188F mutant exhibited similar susceptibility to NVP and DLV, but was more sensitive to EFV inhibition (~4-fold). The representative inhibition curves of NVP versus the mutants are shown in Figure 5. These results are consistent overall with the data obtained from the biochemical assay, and provide unambiguous evidence that aromatic π–π stacking plays an important role in the interactions between the residues and NNRTIs. These are the first set of experimental data that directly assess the importance of π–π stacking in the binding of NNRTIs to the NNRTIBP, by replacing tyrosine with phenylalanine at positions 181 and 188.

## 4. Discussion

The fact that the resistance level of K103N and K103N/Y188F mutant viruses and RTs to the NNRTIs are comparable in both cell-based and biochemical assays strongly suggests that the hydrogen bond between K103N and Y188 may not play an important role in the resistance of K103N substitution to respective NNRTIs. The hydrogen bond between N103 and Y188, observed in the X-ray structure of unliganded K103N RTs, may be interrupted, or significantly weakened, upon the formation of a tertiary complex of K103N RT–primer–template—because it is known that RTs undergo significant conformational changes after binding to the primer and template [28]. Therefore, the results from this study suggest that NNRTIs may have a preference in binding to the tertiary complex rather than to the apo form of RT; hence, diminishing the impact of the hydrogen bond (between the two residues in the apo form of RT) on the affinity of NNRTIs. This hypotheses is supported by finding that the primer grip of the quaternary complex (RT–primer–template–NNRTI) was repositioned away from the active site upon binding to NVP, based on the X-ray structure [28]. If NNRTIs bind to the RT apo form before the RT binds to the primer–template, removal of the hydrogen bonding with K103N/Y188F substitutions should allow the NNRTIs to enter the NNRTIBP of the mutant in a similar fashion to WT; thus regaining the same extent of sensitivity to NNRTIs as the WT. A potential alternative explanation, however, could be that the mutation of K103N/Y188F might induce conformational changes that are distinct from K103N—yet still limit the access of NNRTIBP by NNRTIs—causing the same extent of resistance. 

K103A and K103E mutant viruses displayed similar levels of susceptibility to the NNRTIs, and the respective mutant RTs only showed low levels of resistance (<5-fold) to the NNRTIs in the biochemical assay. These findings suggest that the resistance of K103N (>30 fold) may not be owing to the lower level of hydrophobic interactions by the change of lysine to asparagine (from four carbons to one carbon). Otherwise, by shortening the length of the aliphatic side chain at 103 with K103A or K103E substitution, this should also significantly reduce the binding affinity of NNRTIs to the two mutants to the same extent as to the K103N substitution. Rather, the resistance may come from differences in the electrostatic interactions with NNRTIs between K103N and the WT because the free amino group in lysine is more easily protonated, compared with the amide group in asparagine. Glutamic acid substitution (K103E), on the other hand, is more easily deprotonated under neutral pH conditions. Therefore, the protonated lysine may play a role in the binding of NNRTI to the NNRTIBP. 

Mutation of tyrosine at either the 181 or 188 position either increases or maintains the susceptibility of the mutant viruses to the NNRTIs in both biochemical and cell-based assays. Interestingly, the double mutant Y181F/Y188F uniformly increases the sensitivity to NNRTI inhibition. These results definitely demonstrated the importance of π–π stacking in the binding of the NNRTIs to NNRTIBP. The hydroxyl group of tyrosine may weaken the extent of π–π interactions between the residues and NNRTIs; thus, replacement of tyrosine with phenylalanine tends to improve the potency of NNRTIs via the enhancement of the aromaticity.

In the process of developing cell-based assays using the mutant viruses generated for this study, we found that the replication capacity of the RT variants differs significantly. For example, the K103A and K103E variants displayed better replication capacity than the WT virus. In contrast, the Y181F, Y188F, or Y181F/Y188F mutant reduces the replication capacity significantly—especially the Y181F/Y188F double mutant—which suggests that the hydroxyl group in tyrosine may play a role in the reverse transcription process. These findings are consistent with the relative DNA polymerization rates we observed the in biochemical assay. These results indicate that the residues in NNRTIBP, although not directly involved in DNA polymerization reaction, may contribute to the protein conformations that are important for an efficient reverse transcription.

In summary, results from this study suggest that the hydrogen bond between K103N and Y188 may not play an important role in the resistance of the K103N mutant to NNRTIs. In addition, the aliphatic side chain of lysine may not be involved in the interaction with NNRTIs. Instead, electrostatic interactions could play a role in the resistance of the mutant. This study also provides the first direct evidence on the importance of π–π interactions between the residues of Y181 or Y188 and NNRTIs. As a result, future NNRTIs that are void of aromatic and electrostatic interactions with Y181 and K103 residues, respectively, may offer a better resistance profile compared with current NNRTIs. Furthermore, the residues in NNRTIBP may also contribute to the efficiency of the reverse transcriptase process that, in turn, impacts the replication capacity of the viruses. 

## Figures and Tables

**Table 1:** Inhibitory potency of non-nucleoside reverse transcriptase inhibitors (NNRTIs) (IC_50_, nM) against RT containing different substitutions at the K103, Y181, and/or Y188 positions. *

		RT with K103 Variants					RT with Y181 and/or Y188 Variants						
NNRTI	WT	K103N	K103N/Y188F	K103A	K103E		NNRTI	WT	Y181C	Y188L	Y181F	Y188F	Y181F/Y188F
**EFV**	0.52 ± 0.12	17.0 ± 0.7	10.5 ± 0.4	0.92 ± 0.06	1.37 ± 0.25		**EFV**	0.52 ± 0.12	1.4 ± 0.1	63.0 ± 7.0	0.22 ± 0.02	0.32 ± 0.02	0.17 ± 0.01
**FC**	1.0	33	20	1.8	2.6		**FC**	1.0	2.7	121	0.42	0.62	0.33
**NVP**	140 ± 10	8100 ± 1044	10,033 ± 1861	613 ± 101	687 ± 75		**NVP**	140 ± 10	10,859 ± 822	>25,000	293 ± 12	143 ± 25	54.0 ± 3.5
**FC**	1.0	58	72	4.4	4.9		**FC**	1.0	78	>179	2.1	1.0	0.4
**DLV**	96 ± 14	2600 ± 346	1290 ± 315	170 ± 27	467 ± 55		**DLV**	96 ± 14	1500 ± 200	877 ± 143	78 ± 13	42.0 ± 4.7	12.0 ± 3.5
**FC**	1.0	27	13	1.8	4.9		**FC**	1.0	16	9.1	0.81	0.44	0.13

* The potencies are in means ± standard deviation with more than three replicates. DLV, delavirdine; EFV, efavirenz; FC, fold-change relative to wild type (WT) potency; NVP, nevirapine.

**Table 2:** Susceptibility calculated as the concentration of drugs required to inhibit viral replication by 50% (EC_50_, nM) of HIV-1 RT mutant viruses to NNRTIs. *

		Mutant viruses Containing K103 Mutations in the RT Region							Mutant virus Containing Y181 and/or Y188 Mutations in the RT Region				
NNRTI	WT	K103N	K103N/Y188F	K103A	K103E		NNRTI	WT	Y181C	Y188L	Y181F	Y188F	Y181F/Y188F
EFV	1.2 ± 0.06	69.0 ± 5.7	40 ± 8.1	2.0 ± 0.2	0.9 ± 0.3		EFV	1.20 ± 0.06	1.4 ± 0.1	639 ± 63	0.5 ± 0.07	0.3 ± 0.05	0.4 ± 0.1
FC	1.0	58	33	1.7	0.75		FC	1.0	1.2	533	0.45	0.25	0.33
NVP	95 ± 6	5077 ± 1689	14,500 ± 132	216 ± 51	65 ± 27		NVP	95 ± 6	2269 ± 197	>25,000	37 ± 7	62 ± 11	34.0 ± 2.5
FC	1.0	53	153	2.3	0.68		FC	1.0	24	>263	0.39	0.65	0.36
DLV	26 ± 7	2600 ± 346	1500 ± 43	38 ± 12	64 ± 7		DLV	26 ± 7	1149 ± 301	913 ± 90	8.6 ± 1.3	14 ± 4	4.1 ± 0.12
FC	1.0	100	58	1.5	2.5		FC	1.0	44	35	0.33	0.54	0.16

* The potencies are in means ± standard deviation with more than three replicates.

## References

[1] Palella F.J., Delaney K.M., Moorman A.C., Loveless M.O., Fuhrer J., Satten G.A., Aschman D.J., Holmberg S.D. (1998). Declining morbidity and mortality among patients with advanced human immunodeficiency virus infection. N. Engl. J. Med..

[2] Barth R.E., van der Loeff M.F., Schuurman R., Hoepelman A.I., Wensing A.M. (2010). Virological follow-up of adult patients in antiretroviral treatment programmes in sub-Saharan Africa: A systematic review. Lancet Infect. Dis..

[3] Shahriar R., Rhee S.Y., Liu T.F., Fessel W.J., Scarsella A., Towner W., Holmes S.P., Zolopa A.R., Shafer R.W. (2009). Nonpolymorphic human immunodeficiency virus type 1 protease and reverse transcriptase treatment-selected mutations. Antimicrob. Agents Chemother..

[4] Tambuyzer L., Azijn H., Rimsky L.T., Vingerhoets J., Lecocq P., Kraus G., Picchio G., de Bethune M.P. (2009). Compilation and prevalence of mutations associated with resistance to non-nucleoside reverse transcriptase inhibitors. Antivir. Ther..

[5] Das K., Arnold E. (2013). HIV-1 reverse transcriptase and antiviral drug resistance. Part 1. Curr. Opin. Virol..

[6] Das K., Arnold E. (2013). HIV-1 reverse transcriptase and antiviral drug resistance. Part 2. Curr. Opin. Virol..

[7] Hsiou Y., Ding J., Das K., Clark A.D., Boyer P.L., Lewi P., Janssen P.A., Kleim J.P., Rosner M., Hughes S.H. (2001). The Lys103Asn mutation of HIV-1 RT: A novel mechanism of drug resistance. J. Mol. Biol..

[8] Ding J., Das K., Moereels H., Koymans L., Andries K., Janssen P.A., Hughes S.H., Arnold E. (1995). Structure of HIV-1 RT/TIBO R 86183 complex reveals similarity in the binding of diverse nonnucleoside inhibitors. Nat. Struct. Biol..

[9] Lindberg J., Sigurdsson S., Lowgren S., Andersson H.O., Sahlberg C., Noreen R., Fridborg K., Zhang H., Unge T. (2002). Structural basis for the inhibitory efficacy of efavirenz (DMP-266), MSC194 and PNU142721 towards the HIV-1 RT K103N mutant. Eur. J. Biochem..

[10] Udier-Blagovic M., Tirado-Rives J., Jorgensen W.L. (2004). Structural and energetic analyses of the effects of the K103N mutation of HIV-1 reverse transcriptase on efavirenz analogues. J. Med. Chem..

[11] Feng M., Sachs N.A., Xu M., Grobler J., Blair W., Hazuda D.J., Miller M.D., Lai M.T. (2016). Doravirine Suppresses Common NNRTI-associated Mutants at Clinically Relevant Concentrations. Antimicrob. Agents Chemother..

[12] Ding J., Das K., Tantillo C., Zhang W., Clark A.D., Jessen S., Lu X., Hsiou Y., Jacobo-Molina A., Andries K. (1995). Structure of HIV-1 reverse transcriptase in a complex with the non-nucleoside inhibitor alpha-APA R 95845 at 2.8 A resolution. Structure.

[13] Hogberg M., Sahlberg C., Engelhardt P., Noreen R., Kangasmetsa J., Johansson N.G., Oberg B., Vrang L., Zhang H., Sahlberg B.L. (1999). Urea-PETT compounds as a new class of HIV-1 reverse transcriptase inhibitors. 3. Synthesis and further structure-activity relationship studies of PETT analogues. J. Med. Chem..

[14] Hopkins A.L., Ren J., Esnouf R.M., Willcox B.E., Jones E.Y., Ross C., Miyasaka T., Walker R.T., Tanaka H., Stammers D.K. (1996). Complexes of HIV-1 reverse transcriptase with inhibitors of the HEPT series reveal conformational changes relevant to the design of potent non-nucleoside inhibitors. J. Med. Chem..

[15] Kohlstaedt L.A., Wang J., Friedman J.M., Rice P.A., Steitz T.A. (1992). Crystal structure at 3.5 A resolution of HIV-1 reverse transcriptase complexed with an inhibitor. Science.

[16] Ren J., Esnouf R., Garman E., Somers D., Ross C., Kirby I., Keeling J., Darby G., Jones Y., Stuart D. (1995). High resolution structures of HIV-1 RT from four RT-inhibitor complexes. Nat. Struct. Biol..

[17] Ren J., Diprose J., Warren J., Esnouf R.M., Bird L.E., Ikemizu S., Slater M., Milton J., Balzarini J., Stuart D.I. (2000). Phenylethylthiazolylthiourea (PETT) non-nucleoside inhibitors of HIV-1 and HIV-2 reverse transcriptases. Structural and biochemical analyses. J. Biol. Chem..

[18] Smerdon S.J., Jager J., Wang J., Kohlstaedt L.A., Chirino A.J., Friedman J.M., Rice P.A., Steitz T.A. (1994). Structure of the binding site for nonnucleoside inhibitors of the reverse transcriptase of human immunodeficiency virus type 1. Proc. Natl. Acad. Sci. USA.

[19] Das K., Ding J., Hsiou Y., Clark A.D., Moereels H., Koymans L., Andries K., Pauwels R., Janssen P.A., Boyer P.L. (1996). Crystal structures of 8-Cl and 9-Cl TIBO complexed with wild-type HIV-1 RT and 8-Cl TIBO complexed with the Tyr181Cys HIV-1 RT drug-resistant mutant. J. Mol. Biol..

[20] Ren J., Nichols C., Bird L., Chamberlain P., Weaver K., Short S., Stuart D.I., Stammers D.K. (2001). Structural mechanisms of drug resistance for mutations at codons 181 and 188 in HIV-1 reverse transcriptase and the improved resilience of second generation non-nucleoside inhibitors. J. Mol. Biol..

[21] Shaw-Reid C.A., Munshi V., Graham P., Wolfe A., Witmer M., Danzeisen R., Olsen D.B., Carroll S.S., Embrey M., Wai J.S. (2003). Inhibition of HIV-1 ribonuclease H by a novel diketo acid, 4-[5-(benzoylamino)thien-2-yl]-2,4-dioxobutanoic acid. J. Biol. Chem..

[22] Lu M., Ngo W., Mei Y., Munshi V., Burlein C., Loughran M.H., Williams P.D., Hazuda D.J., Miller M.D., Grobler J.A. (2010). Purification of untagged HIV-1 reverse transcriptase by affinity chromatography. Protein Expr. Purif..

[23] Munshi V., Lu M., Felock P., Barnard R.J., Hazuda D.J., Miller M.D., Lai M.T. (2008). Monitoring the development of non-nucleoside reverse transcriptase inhibitor-associated resistant HIV-1 using an electrochemiluminescence-based reverse transcriptase polymerase assay. Anal. Biochem..

[24] Lai M.T., Lu M., Felock P.J., Hrin R.C., Wang Y.J., Yan Y., Munshi S., McGaughey G.B., Tynebor R.M., Tucker T.J. (2010). Distinct Mutation Pathways of Non-Subtype B HIV-1 during in Vitro Resistance Selection with Non-Nucleoside Reverse Transcriptase Inhibitors. Antimicrob. Agents Chemother.

[25] Lai M.T., Munshi V., Touch S., Tynebor R.M., Tucker T.J., McKenna P.M., Williams T.M., DiStefano D.J., Hazuda D.J., Miller M.D. (2009). Antiviral activity of MK-4965, a novel nonnucleoside reverse transcriptase inhibitor. Antimicrob. Agents Chemother..

[26] Lai M.T., Feng M., Falgueyret J.P., Tawa P., Witmer M., DiStefano D., Li Y., Burch J., Sachs N., Lu M. (2014). In vitro characterization of MK-1439, a novel HIV-1 nonnucleoside reverse transcriptase inhibitor. Antimicrob. Agents Chemother..

[27] Lu M., Felock P.J., Munshi V., Hrin R.C., Wang Y.J., Yan Y., Munshi S., McGaughey G.B., Gomez R., Anthony N.J. (2012). Antiviral activity and in vitro mutation development pathways of MK-6186, a novel nonnucleoside reverse transcriptase inhibitor. Antimicrob. Agents Chemother..

[28] Das K., Martinez S.E., Bauman J.D., Arnold E. (2012). HIV-1 reverse transcriptase complex with DNA and nevirapine reveals non-nucleoside inhibition mechanism. Nat. Struct. Mol. Biol..

